# Needs and challenges of daily life for people with Down syndrome residing in the city of Rome, Italy

**DOI:** 10.1111/j.1365-2788.2011.01432.x

**Published:** 2011-08

**Authors:** M Bertoli, G Biasini, M T Calignano, G Celani, G De Grossi, M C Digilio, C C Fermariello, G Loffredo, F Luchino, A Marchese, S Mazotti, B Menghi, C Razzano, C Tiano, A Zambon Hobart, G Zampino, G Zuccalà

**Affiliations:** 1 Genetica Medica, Ospedale San Pietro FatebenefratelliRoma, Italy; 2 Associazione Culturale PediatriItaly; 3 Coordinamento Nazionale Associazioni Persone con sindrome di DownRoma, Italy; 4 Associazione Italiana Persone con sindrome di DownRoma, Italy; 5 Genetica Medica, Ospedale Pediatrico Bambino GesùRoma, Italy; 6 Comitato Siblings RomaItaly; 7 Fondazione Italiana Verso il FuturoRoma, Italy; 8 Servizio Studi Socio-EconomiciComune di Roma, Ufficio di Statistica, Roma, Italy; 9 Dipartimento di Pediatria, Università Cattolica Sacro CuoreRoma, Italy; 10 Dipartimento di Geriatria, Università Cattolica Sacro CuoreRoma, Italy

**Keywords:** carers, communication, Down syndrome, genetics, intellectual disability, learning disability

## Abstract

**Background:**

Population-based surveys on the quality of life of people with Down syndrome (DS) are difficult to perform because of ethical and legal policies regarding privacy and confidential information, but they are essential for service planning. Little is known about the sample size and variability of quality of life of people with DS living in the city of Rome, which has a population of 2.7 million inhabitants. The aim of the present study is to explore the needs and challenges in health, social integration and daily life, of people with DS living in Rome.

**Methodology:**

A cross-sectional, census-based survey was conducted in 2006. All family doctors (3016 in total) of the National Health Service were involved by the Statistical Bureau of the Municipality of Rome. As per the census, every resident citizen is registered with a family doctor and every person with disabilities is coded. Associations for Down Syndrome encouraged their members to participate in the research. Questionnaires were completed by families of people with DS, in accordance with privacy laws.

**Findings:**

An initial survey, conducted via a letter and a telephone contact with family doctors, identified 884 people with DS residing in the city of Rome. Data on the medical and social conditions of 518 people with DS, ranging in age from 0 to 64 years, were collected. Some 88% of these were living with their original family; 82.1% had one or more siblings, and 19.5% had lost one or both parents. A full 100% of children with DS were enrolled in the public school system. This ensures that they are fully occupied and entirely integrated in society. After secondary school there is a lack of opportunities. Thus, only 10% of adults were working with a regular contract. A mere 42.2% of people with DS aged 25–30 were involved in some form of regular activity (although not always on a daily basis). After the age of 30, the percentage of people demonstrating decline in function increased sharply, while disability-related support decreased. In other words, as people with DS age, daily life evolves increasingly around the home, with only occasional outdoor activities.

**Conclusion:**

The health, employment and social needs of the majority of people with DS in the city of Rome are not being met. The findings of this study underscore the urgent need for more comprehensive inclusion in society of adults with DS and for the provision of support services to create an enabling environment for inclusion. Because of the variability of performance among individuals with DS, there is a need to create more case-specific options in terms of work, living arrangements, social networking and medical services. Schooling and social inclusion in childhood alone do not guarantee a satisfactory quality of life in adulthood. It is argued herewith that policy of inclusion and support should extend over the entire lifetime of people with DS.

## Introduction

### Background

In Italy, parents keep their children with Down syndrome (DS) in the family. Legislation dating back to the 1970s abolished special schools and nursery schools for children with disabilities. Inclusion in mainstream schools has been in effect since 1971. Special classes were abolished by law in 1977. However, following secondary school, suitable services for people with DS are sorely lacking. Most ageing parents continue to take care of their children with DS at home. The issue is whether living at home is appropriate to the multiple needs of DS individuals from birth to senescence, as often it appears that there is little outside support. Further, there are real challenges to what is often referred to as inclusive community living, while quantitative and qualitative assessments of the resources needed to address these challenges are inadequate.

Studies on the quality of life and the state of health of people with DS based on population data are rare. More attention has been paid to children and adolescents with DS than to adults. Besser *et al*. (2007) have estimated the prevalence of children with DS in the 0 to 19 age group born in Atlanta between 1979 and 2003. The number of children living with DS was not determined by actual tracing of living children, but by cross-checking medical records, state vital records and the National Death Index, in order to compensate for underestimation due to the death certificates. Shin *et al*. (2009) expanded the evaluation of DS to prevalence at 19 years of age in 10 regions of the USA. Schieve *et al*. (2009) provided population-based estimates of recent medical conditions for 146 non-institutionalised children aged 3–17 with DS living in Atlanta. Conditions were ascertained through parental reporting. Medical problems were found to be higher among children with DS than among the general population of US children. However, the National Health Interview Survey did not include questions to assess whether children with DS were in separate classrooms or mainstreamed into regular classrooms. Yam *et al*. (2008) evaluated medical records in seven hospitals in Hong Kong, collecting information on the state of health of 407 patients, aged between 0 and 17 years. Prevalence of medical problems was high in children and teenagers with DS and consistent with previous findings.

There is still little information on the conditions of life in adulthood and senescence among people with DS. A number of medical conditions can arise in adults with DS, leading to additional, but preventable, burdens of secondary disability (Chicoine *et al*. 1994; Smith 2001; Yang *et al*. 2002; Roizen & Patterson 2003; Henderson *et al*. 2007; Kerins *et al*. 2008; Rasmussen *et al*. 2008; Yam *et al*. 2008). Guidelines recommend monitoring diseases that may occur in adulthood causing discomfort, and changes in behaviour that can easily be mistaken for early symptoms of Alzheimer disease (Cohen 1999; American Academy of Pediatrics 2001; Italian National Institute of Health 2007). The gradual neglect of health checks leads to delay in diagnosis of physical conditions that contributes to the loss of acquired skills. Henderson *et al*. (2007) evaluated the primary medical records of 89 adults with DS living in Newcastle, UK. These showed that most do not have access to regular healthcare checks, despite the high frequency of common medical complications in adult life. In a 19-year longitudinal study in the USA which began in 1988, Esbensen *et al*. (2007) examined a sample of 169 adults with DS (mean age 33 years), all of whom lived in the parental home. Persons with other intellectual disability (ID) aetiologies served as the control group. Of the original sample, 41 individuals with DS were deceased as of July 2007, most having died of cardiovascular disease or pneumonia. After the age of 35, the annual mortality rate for adults with DS doubled every 6.3 years, compared with 9.6 years for adults with other IDs. As Torr *et al*. (2010) reported, despite the increasing life expectancy of people with DS, these gains started to plateau in the last two decades. In actual fact, mortality increases sharply after the age of 40 for people with DS.

As parents age along with their DS children, they need more support, given that they are concerned for the health of the entire family, as well as the social life of their adult disabled child and his/her long-term living arrangements (Jokinen & Brown 2005). The concomitant ageing of parents and children should be addressed by medical and social services as a problem of the family as a whole, as Jokinen and Brown conclude.

When studies have concentrated on skill development in people with DS, there has usually been a consistent improvement in skills in the early years, followed by a slowing down in development and subsequent change in self-care skills, communication skills and adaptive behaviour in midlife. Rasmussen & Sobsey (1994) conducted a longitudinal analysis of institutionalised adults with and without DS, showing a pattern of decline in self-help and communication skills in several individuals with DS older than 40. In a 3-year prospective longitudinal study of 128 adults with DS, Prasher *et al*. (1998) found that, when the total number of people was evaluated, there was evidence of decline in functional abilities. But when they were divided into three groups – those with medical problems, those with dementia and those who were healthy – the last group showed stability in patterns of function. Oliver *et al*. (1998) assessed 76 people with DS aged 30 years or older, in a 4-year longitudinal study. Severe cognitive deterioration was evident in 28.3% of those aged over 30 and a higher prevalence of impairment was associated with older age. Määttä*et al*. (2006) evaluated 129 people with DS in a retrospective population-based survey drawing on social and healthcare records in Finland. Dates of birth ranged between 1933 and 2003. Records contained data between 1970 and 2004, ranging in age from 0 to 66. People under the age of 20 years were found to have mild or moderate ID more often (71%) than those evaluated at the age of 20 to 39 years (44%) or after the age of 40 years (33%). Nearly 20% of adults did not possess verbal abilities, 44% used at least short sentences, while 35% used single spoken words. There was no mention of their residential situation, and the methodology of data collection was rather confounding. Carr (2008) followed the same cohort of 54 people with DS born in 1964 in South London, UK, describing their everyday life from infancy to 21 years of age, and again at 30, 35 and 40 years. From the outset of the study, all the babies were brought up in their own homes, apart from six who were placed in foster homes. At age 40, 34 people were still in the study and 50% were still living at home. At 35 years old, 44% spent each weekday at the social education centre, but, at age 40, this proportion was reduced to 21%. Daily activities such as cooking, household and sports at 40 years were still similar to those reported previously at 30 years of age: 69%, 88%, 44% respectively. More than one-fourth were never left alone at home for more than 30 min at ages 30 and 40. It was found that limitations in autonomy translate into limitations in social life: the lack of friends was identified as a problem. The author concludes that a wide range of activities and opportunities for social interaction should be made available to people with DS.

Esbensen *et al*. (2008) conducted a 12-year longitudinal study in Massachusetts and Wisconsin, USA, on a voluntary sample of 150 adults with DS, who were part of a group of 461 adults with ID and their ageing parents, all living in the parental home when the study began in 1988. However, during the study period, about a third of the sample moved away from the parental home. Comparisons were made with adults who had ID due to other causes. At the start of the study, 84% of the adults with DS were younger than 40 years of age (mean age was 32) and mothers were between 55 and 85 years of age. Mothers remained the primary respondent throughout the study, even after placement. When the mother died, another family member became the new respondent. Functional abilities such as housekeeping, personal care, meal-related activities and mobility exhibited patterns of change over the 9 years, with ample evidence that developmental change is affected by a variety of environmental factors. These include selected aspects of the family environment, the social setting, opportunities available to individuals and the appropriateness of supportive services. Scales of Independent Behaviour were used at each control. Parental death did not predict differences in any measure of functional ability, but was associated with elevations in all measures of behaviour problems. Being placed outside the home during the study was associated with significant gains in housekeeping, and meal-related activities, but with declines in personal care and health. The age of 40 appears to be a turning point for these individuals, with an elevated risk of health, functional and cognitive problems.

Brown (1998) devoted his work to identifying individualised factors that affect the quality of life of people with ID, and in particular DS. His pilot qualitative research examined the quality of life of people with DS aged 45–70 years (Brown *et al*. 2001). Using a questionnaire, life experience and perceptions were explored, including personal views on the ageing process. Brown & Brown (2003) introduced the concept of family quality of life, as the meeting point of the quality of life of individual members and factors that affect the whole family. Brown *et al*. (2006) explored family quality of life in three types of families: those with a child with DS, those with a child with autism and those of similar household composition but without a child with a disability. Brown *et al*. (2009) showed how all aspects of an individual's life are linked together and how changes in one area may have an effect in another. Their work demonstrates how the needs of ageing adults change over their lifespan. Recommendations are made particularly in relation to the urgency of recognising the central roles of variability, perception and choice, while providing support to encourage dignified and active lifestyles.

Given the ageing of both the carer and adult population with ID, Eley *et al*. (2009) explored the accommodation needs and perceptions of future lifestyle issues in Australia from the perspective of both the carers and the adults with ID. They found: ‘a lack of suitable, available, supported accommodation for people aged 18 years and older with intellectual disabilities. Ageing parents caring at home have little choice but to continue in their caring role’. Burton-Smith *et al*. (2009) investigated the service provision and support needs of Australian family carers. The most frequently endorsed issues were quality day support, respite services and out-of-home accommodation. Bigby (2010) compared five liberal welfare countries: Australia, Canada, Ireland, the UK and the USA. She found that despite consistent identification of similar broad policy issues and overarching goals, little progress has been made in quality day support, respite services and out-of-home accommodation. Felce *et al*. (2008) compared fully staffed group homes and semi-independent living in the UK. On balance, semi-independent living offered certain cost-effective lifestyle advantages, provided that sufficient attention was given to health, living and financial well-being.

Werner *et al*. (2009) investigated family quality of life in 16 families with a member with an ID and multiple diagnoses (with additional conditions, such as behavioural problems), before and after residential placement. Findings suggest that an out-of-home residential placement of a family member with an ID impacts the entire family both positively and negatively. The authors propose a number of recommendations to enhance the quality of life of the individual with a disability, and of the family as a whole. Brown (2004), Cuskelly *et al*. (2006) and Brown *et al*. (2008), and recently Brown *et al*. (2010) examined the international literature and compared support organisations in different countries, describing enormous variability in the degree of disability among people with DS, both in personal skills and level of need, as well as in the quality of social/cultural organisation service provision. They concluded that social isolation and the lack of health checks are likely to result in a more rapid functional decline among people with DS. It is therefore plausible to argue that without the right support, many people who could live a long, serene and dignified life will rapidly compromise their health and quality of life, creating situations that require highly complex care.

### Aim of the present study

The aim of the present study is to explore needs and challenges in health, social and daily living of people with DS living in Rome. Census-type studies, based on the entire population, are difficult to conduct, but they are necessary to tailor services to real needs. The study aimed to capture all people with DS residing in the city of Rome, in a cross-sectional, census-based survey. The study was conducted in 2006 by the City of Rome Bureau of Statistics, Socio-Economic Research Department (Comune di Roma 2007), in collaboration with the Fondazione Italiana Verso il Futuro (http://www.casaloro.it), the AIPD [Associazione Italiana Persone con sindrome di Down – Rome section (http://www.aipd-roma.it)], the Group of Siblings (http://www.siblings.it) and a medical group with an interest in DS (http://www.conosciamocimeglio.it). A first discussion of the findings was presented to Italian paediatricians in 2009, in order to focus attention on DS-related lifespan issues (Luchino *et al*. 2009).

## Methodology

### The questionnaire

A questionnaire was developed for the persons with DS when possible, or for family members, live-in professionals or tutors. After several trials, a final version was formulated and approved by the Statistical Bureau of the Municipality of Rome with the involvement of the entire research team, consisting of sociologists, physicians, psychologists and family members, including parents, siblings and a young woman with DS who was at the time of the survey in a transition phase in terms of living arrangements, between the parental home and a community home. A number of sources were used for the development of the questionnaire, including previous National Institute of Statistics (ISTAT) household surveys (Italian National Institute of Statistics 1999–2000a) as the starting point. The work of Brown on the quality of life of DS individuals was deemed essential. For this reason, it was translated into Italian for dissemination to interested persons who do not speak English (Brown 1998; Brown *et al*. 2001).

The questionnaire contained 47 questions, divided into five sections and themes: structural data on the people with DS and their families, health conditions, social and family daily activities, including school work and residential set-up, self-help, and perceived level of support. The ages of the parents at the time of birth of the child with DS were requested, as was the education level, both of people with DS and of siblings and parents. Many questions were answered on a 4-point scale (Often, Sometimes, A little, Never), and focused on social and family daily activities, such as study, work, vocational or educational activities, sports, taking a walk, going out with friends, church activities, house chores, playing games, watching television and going to the cinema or theatre. Other questions related to areas of self-help, including washing, preparing simple meals, going out alone and using the bus or subway. Verbal communication or ability to make oneself understood by others, competence in writing, reading and use of money, were also addressed, as skills in self-help, communication, writing and reading may affect the degree of autonomy and determine, in part, social relationships. Such questions are not necessarily highly scientific and could be considered rather superficial. Nevertheless, they were included because of their ease of use to people of all cultural backgrounds and educational levels who completed the questionnaires. In fact, their main purpose was to demonstrate the great variability in support services needed by people with DS as well as the variability of the burden of care they present to their families. Answers to such questions used a 5-point scale relating the ‘level of difficulty’ (No difficulty, A little, Some, Great, Complete). One question asked respondents to indicate which operator was closest to them during the life cycle (one could indicate more than one): family doctor, social worker, therapist, teacher, an association, assistants who support people with DS in community housing, volunteer, other. Another question pertained to the perceptions of the support received by families (4-point scale: A great deal, Some, A little, None): ‘How much support do you feel you receive from family members, schoolfellows, neighbours, colleagues, friends, volunteers, associations, social services, or public administration?’. At the end of the questionnaire there was space for narrative and other comments.

It is important to note that each item had been agreed in substance and form by the entire joint team of professionals and family members involved in the research.

The questionnaire form is on the site of the Municipality of Rome (Comune di Roma 2007): http://www.comune.roma.it/was/repository/ContentManagement/information/P488537847/NoiAltricop.pdf (pages 159–170), and http://www.conosciamocimeglio.it/Down/docs/2007/NoiAltricop.pdf

### Data collection

Rome has a population of about 2.7 million inhabitants and is divided in 19 municipalities (http://www.comune.roma.it/wps/portal/pcr?jppagecode=municipi.wp) with a wide range of organisations and resources. The census was based on the fact that every resident is assigned to a family doctor and each person with a disability is given a code. A letter was sent to all family doctors (3016 in total) in the city of Rome, including 406 family paediatricians and 2610 general practitioners. Subsequently, telephone contact was made with each doctor, asking the question: ‘What is the presence or absence among your patients of patients with DS?’. All data relating to contacts and responses were recorded. Doctors who responded that they had patients with DS were hand-delivered the questionnaires, along with a letter of introduction to the families. The doctors handed these to concerned families and then collected the completed questionnaires in sealed envelopes, which were later collected in person by skilled Civil Service Volunteers, who were graduates in Statistics or Sociology. The Municipality of Rome then addressed a request to family organisations (the Associazione Italiana Persone con sindrome di Down – AIPD – Section of Rome, the Fondazione Italiana verso il Futuro and the Siblings Group), in order to facilitate contact with concerned families. These associations asked their members to complete and send the questionnaire directly to the Municipality of Rome in a sealed envelope, if this had not already been done by the doctors.

The research was carried out in three phases over 3 years:

*Phase 1*: September 2004–December 2005: preliminary analysis and planning, identification of study objectives, design of survey methodology and detection instrument. The list of doctors was received by the administrative office in October 2004.

*Phase 2*: January 2006–September 2006: assignment of volunteers by the Ministry of Social Welfare, inception of the execution phase of the survey, with extensive field work to collect data.

*Phase 3*: September 2006–September 2007: data processing, quality control and analysis of the findings.

## Main findings

Table 1 describes the methodology and quality of data collection. During the first contact established by general practitioners and family paediatricians, 884 people with DS residing in the city of Rome were counted. At the end of the survey, 518 completed questionnaires were collected.

**Table 1:** Data collection methodology and data quality

***n***	
3016	Total number of doctors included in the list affiliated to the National Service, October 2004 (406 paediatricians and 2610 general practitioners)
236	Doctors not traced in January 2006 (change of telephone number, address, consulting room hours, transferred, retired or deceased)
2780	Total number of doctors contacted by phone
97	Doctors who refused to cooperate
1981	Doctors who said that they had no patients with Down syndrome
702	Doctors contacted who said that they had one or a number of patients with Down syndrome who cooperated by delivering and collecting the completed questionnaires from the families
884	People with Down syndrome counted by the telephone survey
884	Questionnaires delivered to doctors for distribution to the families
347	Returned completed questionnaires at the first collection after 8 weeks
144	Questionnaires sent to the city of Rome after reminder made by families associations
27	Questionnaires delivered to the secretary of AIPD – section of Rome in a sealed envelope
518	Total number of questionnaires completed by the end of the survey (58.6% of 884)

The questionnaire was completed in 9.2% of cases by the person with Down syndrome, in 31% of cases by fathers, 57.1% by mothers, 20.6% by siblings, 3.1% by other family members (some grandparents), in 3.3% of cases by a social worker and in 8.8% of cases by ‘others’.

AIPD, Associazione Italiana Persone con sindrome di Down.

Table 2 describes the distribution by age and sex of people with DS who answered the questionnaire. Among the 518 people with DS (282 male, 236 female) who completed the questionnaire, 80 were children (0–10 years old), 101 were 11–19 years old, 93 were 20–30 years old, 91 were 31–40 years old, 77 were 41–50 years old, and 51 were 51–64 years old, of which 11 were over 60. The two eldest were 64 years old. Twenty-five people did not state their age.

**Table 2:** Distribution by age and gender

	**Male**	**Female**	**Total**
Age group*	***n***	***n***	***n* (%)**
0–2	12	7	19 (3.7)
3–5	11	13	24 (4.6)
6–10	23	14	37 (7.1)
11–13	17	22	39 (7.5)
14–19	38	24	62 (12)
20–24	17	31	48 (9.3)
25–30	25	20	45 (8.7)
31–35	24	23	47 (9.1)
36–40	22	22	44 (8.7)
41–45	20	15	35 (6.8)
46–50	25	17	42 (8.1)
51–55	18	10	28 (5.2)
56–64	14	9	23 (4.6)
Age not declared†	16	9	25 (4.6)
Total	282	236	518 (100)

* Age group adjusted to compare with Italian school ages (Nursery, Kindergarten, Elementary, Middle, High), see Table 6.

† No adjustment was made when age was not declared for privacy reasons.

A brief summary of demographic data not shown in the tables is presented below:

Only 10% of the people interviewed were employed with a regular contract, with no differences between male participants and female participants. Some 88% were living with their families, 4.1% in small community family houses, and 6.9% in large establishments. Maternal age at birth of the child with DS was asked: 49% of births occurred when the mother was less than 35 years of age. The ages of parents at the time of the survey (2006) were also asked: some 16% of people with DS had a mother aged 55–64, 17% had a mother of 65–74 years old, and 15.6% a mother older than 75 years. Mothers had a high school degree in about 30% of cases and a university degree in about 12.5% of cases, while general population data in 2001 reported respectively 32% and 7%. A total of 14 (2.7%) persons with DS had experienced the death of their mother, 49 (9.4%) the death of their father and 38 (7.3%) the death of both parents. Information about siblings showed that 82.1% of the respondents had a brother or sister: 45.8% had only one, 27.6% had two, and 8.7% had three siblings. About 50.3% of the people with DS in the study had siblings living with them.

Table 3 describes the distribution of data collected in the 19 municipalities of Rome.

**Table 3:** Territorial distribution of citizens with Down syndrome (DS) who answered the questionnaires

Municipality*	**Population**	**Citizens with DS who answered the questionnaires**	**Citizens with DS who answered the questionnaires/10 000**†
I	122 574	19	1.5
II	124 545	20	1.6
III	57 378	7	1.2
IV	206 752	34	1.6
V	186 869	37	2
VI	133 851	25	1.8
VII	126 875	31	2.4
VIII	194 315	43	2.2
IX	134 937	18	1.3
X	181 571	31	1.7
XI	140 079	14	1
XII	156 871	24	1.5
XIII	188 183	16	0.8
XV	155 641	14	0.9
XVI	148 667	28	1.9
XVII	75 601	9	1.2
XVIII	134 165	29	2.2
XIX	179 079	22	1.2
XX	145 399	16	1.1
Municipality not declared‡		81	

* Municipality XIV (Fiumicino) is absent because it has become independent of Rome.

† If the population over 65 years is removed (552 431 people: see Table 4), medium prevalence of all 518 people with DS who answered to the questionnaire is 2.3/10 000.

‡ To respect the privacy, no adjustment was made when the municipality was not declared, even if it was possible to deduce it from cross-checking of data.

Table 4 compares age group DS prevalence estimates in Atlanta 2003 (Besser *et al*. 2007) with age group prevalence of respondents to the questionnaire in the present Rome 2006 survey. The number of expected cases in Rome was calculated following Besser's estimate prevalence by age group, from 0 to 19 years, applied on the demographic landscape of Rome. In the age groups 0–4 years and 5–9 years, the survey captured respectively 28.8% and 32.7% of the expected number, while for ages 10–14 and 15–19 it captured 67% and 79% of expected cases. No data are available to compare older age groups. (Tables 1–4 show the absolute numbers of cases of people with DS. Elsewhere, only percentages are shown so as not to allow the cross-checking of territorial and qualitative data for privacy reasons.)

**Table 4:** Age group prevalence* estimate in Atlanta 2003 (Besser *et al*. 2007), compared with age group prevalence of respondents to the questionnaire in the present cross-sectional survey in Rome, 2006

**Atlanta 2003. Estimated Down syndrome prevalence**			
	
**Age group**	**Down syndrome cases estimated**	**Population**	Prevalence*
0–4	269	245 545	10.96
5–9	201	219 744	9.15
10–14	152	223 474	6.8
15–19	116	202 438	5.73

**Rome 2006. Questionnaires collected**			
	
**Age group (5-year intervals)**	**Questionnaires collected (*n*)**	**Population**	Prevalence*
0–4	38	120 709	3.14
5–9	35	117 693	2.98
10–14	55	120 665	4.56
15–19	53	117 018	4.52
20–24	48	132 739	3.61
25–29	36	183 176	1.95
30–34	46	234 770	1.95
35–39	43	254 971	1.68
40–44	43	230 158	1.86
45–49	36	199 907	1.8
50–54	31	183 180	1.69
55–59	18	183 816	0.97
60–64	11	179 698	0.61
65–69	–	163 754	–
70–74	–	140 528	–
75–79	–	110 667	–
80–84	–	76 667	–
Over 84	–	60 815	–

**Number of questionnaires collected compared with number expected in Rome, following Besser prevalence estimates**			

**Age group**	**Questionnaires collected, Rome 2006**	***n* expected in Rome, based on Atlanta 2003 estimate prevalences**	***n* of questionnaires/expected cases (%)**
0–4	38	132	28.8
5–9	35	107	32.7
10–14	55	82	67
15–19	53	67	79

* Point prevalence per 10 000 population.

Table 5 describes frequencies of associated diseases at the time of the survey. It is noteworthy, although not reported in the table, that 7.3% reported no associated disease. Some 17% reported only one pathology, while 18.7% reported two pathologies and 51% reported three or more associated diseases. Regular health checks were received by 100% of children aged 5 years, by 80% of people at 19 years, by 50% at 35 years and by 60% at over 50 years. The educational level of the mother was higher among those who had health checks than among those who did not.

**Table 5:** Diseases, at the time of the survey, as reported in the questionnaires, by gender (%)

	**Gender**		**Total**
		
**Diseases**	**Male**	**Female**	**%**
Congenital heart disease	16.6	21.6	19
Cardiovascular diseases (other)	4.6	5.6	5.1
Hearing loss	10.4	11.7	11
Eye diseases	49.4	42.9	46.3
Dental diseases	46.3	49.8	48
Diabetes	1.9	2.6	2.2
Thyroid	20.5	31.2	25.5
Celiac disease	3.1	5.2	4.1
Dermatology	25.1	26	25.5
Orthopaedics	28.6	33.3	30.8
Overweight/obesity	24.7	37.7	30.8
Tumours	1.5	0.9	1.2
Neurology	13.9	11.7	12.9
Psychiatry	11.2	7.8	9.6
Behavioural problems	181	13	15.7
No pathology	10	6.5	8.4

Table 6 shows school attendance by age group. Data show some delay in transition from one stage of schooling to the next (from kindergarten to primary, to middle and high school), and sometimes a long enrolment in high school beyond 19 years of age. Only 2.6% of children with DS leave school at an early age, and 11.3% are no longer in mainstream schools at ages 14–19.

Table 7 shows the extent to which people with DS are engaged in various activities during the week. A comparison between Tables 6 and 7 shows that ‘work’ does not gradually replace the space left empty by school. A maximum of 30% of people with DS of 25–30 years of age are employed. At the bottom of the table, it is shown that the percentage of people listed as ‘never [working] in the week’ constituted about half of those over 20 years of age, rising to 70% after 40 years. Most activities ‘often in the week’ received a score of below 20% among school leavers. Even house chores do not appear to be particularly encouraged. Sports ‘never in the week’ was the less frequent answer, compared with other activities. This can be considered a positive trend and offers hope for the future.

**Table 6:** Schooling by age group (%)

	**Age group**											
	
School*	**0–2**	**3–5**	**6–10**	**11–13**	**14–19**	**20–24**	**25–30**	**31–35**	**36–40**	**41–45**	**46–50**	**Over 50**
No school	36.8	–	–	–	4.8	27.1	68.9	53.2	66.7	74.3	78.6	84.3
Nursery/Kindergarten	57.9	**100.0**	24.3	–	–	–	–	–	–	–	–	–
Elementary school	–	–	73.0	41.0	–	–	–	–	–	–	–	–
Middle school	–	–	–	53.8	21.0	–	–	–	–	–	–	–
High school	–	–	–	2.6	58.1	16.7	2.2	–	–	–	–	–
Vocational courses	–	–	–	–	9.7	31.3	11.1	2.1	4.4	–	2.4	–
Other†	–	–	2.7	2.6	4.8	22.9	13.3	25.6	22.2	17.1	9.5	13.7
Did not answer	5.3	–	–	–	1.6	2.1	4.4	19.1	6.7	8.6	9.5	2.0

* In Italy, mainstreaming starts in Nursery. Kindergarten starts at 3 years old, Elementary School starts at 6, Middle School at 11, High School at 14 (normally it lasts 5 years, but 6 years is common in these cases, given the difficulties in transitioning to adult Down syndrome services).

† ‘Other’: any activities aimed at obtaining or maintaining cognitive ability, public or private, seldom on a daily basis. This item overlaps with the item ‘Educational activities’ in Table 7.

**Table 7:** The table shows how often (*) people with Down syndrome are engaged in various activities during the week, by age group

	**Age group**									
	
	**6–10**	**11–13**	**14–19**	**20–24**	**25–30**	**31–35**	**36–40**	**41–45**	**46–50**	**Over 50**
**‘Often in the week’**										
Work (including sheltered)			8.0	10.4	31.1	19.1	15.6	5.7	11.9	7.8
Educational activities†	8.1	5.1	8.1	27.1	11.1	12.8	13.3	5.7	23.8	15.7
Sports	29.7	43.6	29.0	27.1	31.1	34.0	20.0	2.9	19.0	2.0
Walking	48.6	43.6	40.3	35.4	33.3	48.9	48.9	48.6	54.8	51.0
Out with friends	5.6	5.1	14.5	20.8	20.0	21.3	22.2	11.4	21.4	27.5
Church-parish activities	8.1	15.4	14.5	6.3	15.6	8.5	17.8	17.1	19.0	9.8
Doing house work		5.1	9.7	8.3	15.6	12.8	15.6	20.0	14.3	13.7
Playing games	81.1	92.3	54.8	35.4	24.4	27.7	31.1	20.0	16.7	21.6
Watching television	62.2	79.5	83.9	72.9	68.9	66.0	62.2	51.4	52.4	43.1
Going to the cinema	8.1	5.1	16.1	6.3	4.4	10.6	13.3	5.7	4.8	3.9
Going to the theatre			8.1		2.2	6.4	8.9		4.8	2.0
**‘Never in the week’**										
Work (including sheltered)			72.0	54.2	40.0	53.2	62.2	77.1	66.7	66.7
Sports	24.3	15.4	8.1	16.7	15.6	27.7	40.0	54.3	47.6	66.7

* Each answer was weighted on a 4-point scale (Often, Sometimes, A little, Never).

† Educational activities: any activities to maintain or cultivate cognitive ability (such as crafts, gardening, theatre, music), autonomy and social interaction. This item overlaps with the item ‘Other’ in Table 6.

Table 8 describes some basic self-help skills, distributed by age group. There is a marked difference between the groups under and over 30 years. For example, the age group 25–30 presents no difficulty in ‘preparing simple meals’ in 22.2% of cases and no difficulty in ‘going out alone’ in 68.9% of cases. The next age group, 30–35 years of age, presents the same ease in only 6.4% and 36.2% of cases respectively. Similarly, the use of money (out of table) was common in 68% of cases up to 30 years, but in a mere 30% of cases after age 30.

**Table 8:** Level of difficulty declared in some areas of self-help

	**Age group**							
	
**Level of difficulty**	**14–19**	**20–24**	**25–30**	**31–35**	**36–40**	**41–45**	**46–50**	**Over 50**
**Washes him/herself**								
Complete			4.4	4.3	17.8	17.1	11.9	19.6
Great	4.8	2.1	2.2	10.6	2.2	20.0	9.5	17.6
Some	17.7	6.3	6.7	23.4	17.8	25.7	19.0	23.5
A little	25.8	25.0	28.9	23.4	13.3	14.3	31.0	17.6
No difficulty	50.0	62.5	57.8	38.3	48.9	22.9	26.2	21.6
Did not answer	1.6	2.1					2.4	
**Prepares simple meals**								
Complete	12	25	22.2	48.9	48.9	71.4	64.3	68.6
Great	20	14.6	11.1	10.6	6.7	2.9	7.1	3.9
Some	28	20.8	20	17	13.3	11.4	9.5	5.9
A little	20	18.8	22.2	14.9	13.3	2.9	4.8	9.8
No difficulty	20	16.7	22.2	6.4	11.1	–	4.8	3.9
Did not answer	–	4.2	2.2	2.1	6.6	11.5	9.5	7.9
**Goes out alone**								
Complete	8	25	8.9	23.4	42.2	31.4	40.5	49
Great	–	–	4.4	8.5	4.4	14.3	14.3	13.7
Some	16	14.6	15.6	19.1	13.3	20	7.1	3.9
A little	20	20.8	2.2	10.6	11.1	5.7	2.4	13.7
No difficulty	56	37.5	68.9	36.2	26.7	28.6	28.6	19.6
Did not answer	–	2.1	–	2.1	2.2	–	7.2	–
**Uses bus or metro**								
Complete	20	29.2	31.1	53.2	57.8	77.1	61.9	70.6
Great	8	12.5	6.7	10.6	13.3	8.6	16.7	9.8
Some	20	18.8	13.3	14.9	2.2	2.9	2.4	3.9
A little	32	16.7	13.3	8.5	4.4		2.4	3.9
No difficulty	16	18.8	33.3	10.6	20	5.7	4.8	5.9
Did not answer	4	4.2	2.2	2.1	2.2	5.7	11.9	2

Answers on a 5-point scale (%).

Table 9 indicates the level of difficulty in communication for people with DS aged 14–64 (374 people), as a whole.

**Table 9:** Level of difficulty in communicating

	**Level of difficulty**				
	
	**No difficulty (can do it easily)**	**A little**	**Some**	**Great**	**Complete (cannot do it at all)**
Understands verbal communication	41.6	25	21	6.3	2.4
Uses verbal communication	15.2	24.4	28.5	17.8	7.7
Makes him/herself understood	27.3	25.1	29.5	11.3	3

Age 14–62 (374 people). Answers on a 5-point scale (%).

Table 10 describes variability in writing and reading by age group. The proportion of ‘cannot write at all’ or ‘cannot read at all’ was 30.8% and 23% for the 11–13 years age group, 18.8% and 19.4% for the 20–24 years age group, 28.9% and 6% for the 25–30 years age group. Nevertheless, up to 50% of young adults can write and read without difficulty. A salient observation (out of table) concerns the frequency with which DS people stated listening to music with ease (80%) or listening to somebody reading aloud to them. In the latter activity, 50% responded that they listened with ‘ease’. It would be useful to know whether the other 50% ever had this opportunity.

**Table 10:** Variability in writing and reading, by age group (answers on a 5-point scale*) (%)

	**Age group**									
	
**Write and read**	**6–10**	**11–13**	**14–19**	**20–24**	**25–30**	**31–35**	**36–40**	**41–45**	**46–50**	**Over 50**
**Can do the following easily**										
Write	13.5	30.8	41.9	52.1	48.9	29.8	26.7	14.3	23.8	21.6
Sign own name	29.7	48.7	66.1	68.8	75.6	66	60	28.6	52.4	31.4
Read	8.1	28.2	33.9	52.1	35.6	40.4	40	11.4	28.6	17.6
**Cannot do the following at all**										
Write	37.8	30.8	24.2	18.8	28.9	34.0	40.0	60.0	45.2	58.8
Sign own name	37.8	28.2	16.1	8.3	11.1	17.0	24.4	48.6	31.0	47.1
Read	29.7	23.1	19.4	6.3	17.8	21.3	24.4	54.3	23.8	60.8

* No difficulty (**can do it easily**), A little, Some, Great, Complete (**cannot do it at all**). The table shows only the two extreme responses.

Table 11 indicates the level of perceived support from the social network, the service coordination/social work and public administration. The family has a central role in supporting itself and the person with DS. Answers of ‘not at all’ were particularly common in relation to services and public administration, but also in relation to social networks, which are not perceived as easily accessible. A question (out of table) was asked of two groups of DS children to establish which operators were considered closest in the care pathway. The two groups of children aged 0–2 and 3–5 years indicated social workers and therapists (55% and 37%), paediatricians (66% and 79%) and associations (38% and 20%). The group of children between 10 and 19 years of age singled out the teachers, with a decreasing percentage from kindergarten to high school, from 87% to 64%. Parents' associations were the first point of reference for people aged 20–30 years (46.5%). After the age of 30, the percentages dwindle for each operator to under 10% and only the family doctor maintains a rate of 49% for those over 50 years of age.

**Table 11:** Level of perceived support

	**Level of perceived support**			
	
**Source of support**	**A great deal**	**Some**	**A little**	**Not at all**
Family members	83.6	7.4	2.9	1.5
Schoolfellows, neighbours, colleagues	17.1	28.1	8.1	22.9
Friends	9.8	23.6	22.2	22
Volunteers	6.2	15.4	8.1	43.1
Associations	14.9	17.6	7.7	36.3
Service coordination/social work	8.9	19.9	13.9	37.8
Public administration	3.3	8.7	10.8	38

Answers on a 4-point scale (%).

Table 12 shows the amount of time people with DS spent with parents. The category ‘a great deal’ received very high scores for every age group, from infancy to old age, while time with friends did not increase with age, as would have been desirable. Time spent with siblings (out of table) received very high scores, particularly in infancy, but also throughout the lifespan of people with DS.

**Table 12:** Time spent with friends and time with parents during the week, by age group

	**Age group**											
	
	**0–2**	**3–5**	**6–10**	**11–13**	**14–19**	**20–24**	**25–30**	**31–35**	**36–40**	**41–45**	**46–50**	**Over 50**
**Time with friends**												
A great deal	5.6	25.0	18.9	7.7	1.6	6.3	8.9	14.9	17.8	11.4	23.8	23.5
Some	5.6	29.2	40.5	38.5	27.4	27.1	31.1	21.3	22.2	11.4	23.8	15.7
A little	22.2	12.5	29.7	25.6	50.0	35.4	28.9	34.0	22.2	17.1	11.9	13.7
Not at all	5.6		2.7	15.4	16.1	25.0	22.2	19.1	22.2	34.3	21.4	27.5
Do not know						2.1	2.2	2.1	2.2	2.9		
No answer	61.1	33.3	8.1	12.8	4.8	4.2	6.7	8.5	13.3	22.9	19.0	19.6
**Time with parents**												
A great deal	78.9	83.3	86.5	76.3	83.3	74.4	80.0	73.0	75.0	72.7	73.3	40.0
Some		8.3	13.5	21.1	15.0	25.6	17.5	24.3	16.7	9.1		
A little							2.5		8.3			
Not at all								2.7		9.1		
Do not know											6.7	
No answer	21.1	8.3		2.6	1.7					9.1	20.0	60.0

Answers on a 4-point scale (%).

## Qualitative findings

The questionnaire reserved space for narrative content and general observations. One respondent highlighted the interruption of day services because of her inability to reach them due to severe arthritis and the lack of transport (female, 45 years). Another reported being unable to leave the home because of severe behavioural changes (male, 50 years). The mother of a 30-year-old man with DS, who was among the most active, with regular part-time work, wrote: ‘We spend half of our lives teaching our children how “the others live”, just to tell them: “NO, this is not a choice for you” ’. Another noted: ‘We teach them to go out alone, but then there is no place for them to go’.

Most respondents used the free space to ask for help. The feeling of isolation among families with DS individuals appears to be such that, for older families, a questionnaire sent by the City through the National Health Service was perceived as an unexpected and welcome bridge of communication.

## Discussion

### Methodology of data collection and data quality

One of the limitations of a survey covering the entire population, on such a delicate and private subject, is the collection of data, given privacy laws for health information. The mediation of family physicians of the National Health Service, which is organised on a territorial basis, presented both strengths and weaknesses. Strengths included the following: (1) privacy was guaranteed; (2) people with DS who were not involved with associations were reached; and (3) extensive contact with doctors across the city reached more isolated people as well as those not involved in services.

The main weakness was the occasional non-availability of doctors and families, which led to loss of data.

Another limitation relates to the questionnaire itself: despite extensive preparatory work on the form, some questions were not clear enough and received inconsistent responses, as may occur with detection techniques that use self-completion of the questionnaire without the direct support of survey personnel.

### Prevalence of Down syndrome by age group

The present study went to great lengths to contact all individuals with DS in Rome. The percentage of responses to the questionnaire (518) compared tothe number of people counted in the first telephone interview (884) was 58.60%. It is not possible to ascertain the age distribution of cases that did not respond to the questionnaire, and it is difficult to determine how many cases missed the first contact call. In an attempt to quantify under-detection, findings were compared with those derived from prevalence by age group estimates in Atlanta (Besser *et al*. 2007). The data (Table 4) suggest that under-detection is more concentrated in infancy: about 67% of expected cases were reached with the questionnaire in the age group 11–13, and up to 79% in the age group 15–19. Prevalence estimates in Atlanta (Besser *et al*. 2007) were very carefully based on multiple-source information – both vital and death records – of people diagnosed as DS at birth. Unfortunately, there are no data to compare prevalence of DS in adult age groups.

For adulthood and senescence (Sherman *et al*. 2007), there are no reliable data anywhere in the world: Sherman *et al*. conclude: ‘It is necessary to collect DS prevalence data in the population for different age groups to plan evidence-based health and social services for people with Down syndrome in the different seasons of life.’ These data have never been available because there are no population-based registries beyond infancy (Besser *et al*. 2007).

In view of the above lacunae in DS prevalence data across all age groups, the data collected in this survey offer valuable information and insight into the context-specific challenges of establishing sample size by age group, determining prevalence of DS people living in the city of Rome as the baseline for the design of appropriate interventions.

### Representativeness of the data collected

Certain features specific to the population with DS were taken into account in order to determine whether those interviewed could be considered representative of the people who did not respond to the questionnaire or whether we should hypothesise a selection bias. A large body of literature is available on associated pathology, as mentioned briefly in *Introduction*. Without going into details, most of the percentages of associated diseases were consistent with those reported in the literature for populations with DS, except congenital heart disease, which was strongly under-detected – 19% against 50% expected. It is likely that newborns with severe associated malformation have little contact with the local family paediatrician and more with the specialists in hospital.

An observation in favour of representativeness is the homogeneous distribution of the sample by sex and age group. Spatial distribution seems to be fairly homogeneous. Distribution by instruction level of the mothers was consistent with the level of the general population, as expected, with a slightly higher prevalence of university degree education for those who answered the questionnaires. Note that population data are based on the 2001 national census.

Another point concerns the distribution of maternal age at birth. The questionnaire shows that about 50% of the births occurred when the mother was under the age of 35 (births occurred between 1944 and 2006). This was within the expected range (Morris *et al*. 2003). But the distribution of maternal age at delivery in the general population in Rome has changed significantly over time: in 2009 local demographic data (Di Lallo *et al*. 2009) indicate that the proportion of women older than 34 years rose from 9% in 1982 to 35% in 2009. The possibility of having a child with DS increases with maternal age, but it has been estimated that those who choose prenatal diagnosis in Italy (available since the late 1970s) terminate pregnancy in 93% of cases with positive results (Boyd *et al*. 2008). Despite the increase in maternal age, the proportion of children born to younger mothers does not appear to have changed over time.

Based on the above, it is plausible to argue that the data collected are representative of the DS population living in families in the municipal territory. Nevertheless, this survey does not make reference to adults with DS who live in community settings or institutions, as the number in the sample was too small and we can also assume some emigration due to transfer to institutions outside of the municipal area. It is important to plan further investigations in this direction.

### Lack of support services and decline in function among people with Down syndrome

This study attempted to quantify and qualify a reality that is well known by individual families, but is often invisible to society at large. The absence of data on prevalence, and on the living conditions and quality of life of people with DS inadvertently contributes to the further marginalisation, isolation and vulnerability of these men and women and their families. Further, in the absence of evidence, policy and programming are lagging behind. The present study puts into sharp focus the existing gap of external support services for people with DS after secondary school, which extends well into adulthood.

The data demonstrate that the quality of life in ageing DS people in Rome is very poor, often characterised by limited autonomy, few employment opportunities, a compromised health status and a dearth of social interaction. Writing, reading and communication abilities have been described at a rudimentary level, but reflect the enormous variability of need. There is a large discrepancy between the percentage of people reporting difficulties in verbal communication, as well as in writing and reading, and the percentage of those who claim to listen to being read aloud ‘with ease’. This activity should be offered more systematically, to stimulate the imagination and enhance emotional and cognitive communication, without forcing people with DS to ‘perform’. Society should be sensitised to the fact that through the spoken word, it is possible to reach persons with DS to a much greater extent than is generally assumed. Studies have shown that storytelling to children with DS can result in higher comprehension than formal measures of expressive language would predict (Miles & Chapman 2002). The home literacy environment of pre-school children with IDs, as studied by van der Schuit *et al*. (2009), shows that parents adapt to the developmental level of their child. Reduced linguistic capacity is dangerously equated with a reduced capacity to understand at any age. This can potentially inflict considerable damage to a person with DS, and contribute to an overall decline in function and well-being.

Basic self-help skills need to be prioritised in order to evaluate the burden on the family, and the level of support needed. These data (Table 8) should be taken into account when training service operators. A reduced range of ability among people with DS is reported as early as age 30 in our survey, while in the international literature this does not occur until the age of 40.

The cohort of DS individuals who are now 30–35 years old was possibly the first to have been raised fully integrated in society, in terms of attending normal schools and being involved in a plethora of community and social activities (gym clubs, playgrounds, concerts, etc.). However, after secondary school, this cohort faced a sudden abandonment: support services for adult people with DS and their families are scant and have faced particular challenges, having been instituted with considerable delays. Respondent answers complaining of very little support did not conflict with objective observations. This points to the lack of activities available for different age groups. In fact, after 20 years of age, only 30–40% of people with DS were found to be involved in some work or educational activity. Much of their free time involves little activity. This could well result in a deterioration of their mental function and capacity and their overall well-being. These findings are consistent with those of Werner *et al*. (2009), who worked with persons with ID in Canada.

Sport deserves a special mention in this context: many disabled people do not engage in sports or physical activity in general (Italian National Institute of Statistics 1999–2000b), but for people with DS there is a positive opening for these activities, as shown in the findings. Unfortunately, older adults are not as involved in sports as they should be. It has been shown that it is never too late to start, partly to prevent diseases associated with additional disabilities, with consequent loss of autonomy and the need for additional assistance (Jobling 2001; Sayers Menear 2007; Murphy & Carbone 2008). The mental, social and physical health benefits of sports on the quality of the life of the individual and in economic terms are significant. Physical activity is an investment for society. Rome is full of initiatives supported by AIPD which are accessible to everyone. However, much remains to be done. Policymakers must recognise and support those pilot schemes that encourage DS men and women to participate in sports, from rugby, to judo, dance, swimming and skiing – always alongside normal children.

Figures on the participation of DS people in employment, including sheltered work, are limited. Even calculating the percentage of persons employed in educational activities (such as in day centres, seldom available on a daily basis), it appears that two-thirds of adults with DS have ‘full days of empty time’. ‘The group of study on work inclusion’ of the National Coordination of Family Associations for Down Syndrome (Coordown 2008) collected 1167 questionnaires on training and job placement of adults with DS from associations all over Italy in 2007, including AIPD, UNIDOWN and 30 other associations. The research shows that only 10% of respondents worked with a regular employment contract (updated on 15 March 2007), while 82% were not even engaged in training internships. These data are similar to, and overlap with, those found in the city of Rome in 2006 in the present study. The lack of employment opportunities is likely to contribute to a loss of acquired skills. What is required is a policy of work inclusion for all, on a daily basis. Some people with DS enter the free job market, but most of these adults in the city of Rome urgently require opportunities for daily employment in day centres or sheltered work. Such inclusion is also likely to contribute to improved social interaction, increased autonomy, better health checks and relief for families whose quality of life may be enhanced considerably. Living at home, but with close ties to the community, is likely to enrich the community as well.

The media tends to focus on people with DS who are coping remarkably well. This may be encouraging, but it also contributes to the marginalisation of the majority of people living with DS who face enormous challenges. In particular, a long list of difficult situations arises as people with DS age and develop health or behavioural problems. A randomised trial in Italy (Bernabei *et al*. 1998) showed that integrated social and medical care with case management programmes may provide a cost-effective approach to reducing admission to institutions and functional decline in older people living in the community. The present data, which are limited to the specific circumstances of frailty of DS, reveal a more generalised problem where the most vulnerable do not even have the capacity to make their voices heard. The data also show a real urgency for day support services for those DS adults living in their original family, who are close to senescence, and for residential solutions for those who need them or those who opt in favour of them.

### Conclusions and challenges

The 2006 population-based survey on sample size and quality of life of people with DS living in Rome, although difficult to perform because of ethical and legal policies regarding privacy and confidential information, yielded a wealth of data and information critical for both service planning and service provision. The findings of this survey have put into sharp focus the needs and challenges in health, social integration and daily life of people with DS and their families. These needs are currently not being met. There is an urgent need for inclusion in society of adults with DS and for the provision of appropriate support services. Because of the variability of performance among individuals with DS, individualised options and opportunities are required in terms of work, living arrangements and social networking. Without the right support, many people who could live a long, serene and dignified life gradually compromise their health and quality of life, creating situations that require highly complex care. This article thus argues for evidence-based health and social services for people with DS and their families throughout their life.

It has been shown that in Italy, legislation dating back to the 1970s abolished special schools and nursery schools for children with disabilities. Legislation limits classroom size to 20 children, including those with special needs. Supplementary support teachers and therapists are provided by the local health system. At present, as a result of the financial crisis and related political responses, there are staffing problems across schools because of the progressive reduction in the number of teachers. Thus, minimal support is available in schools for children with disabilities, and more than one disabled child may have to be included in each class. However, after 40 years of inclusive schooling, children with disabilities are socially integrated. Inclusive schooling and social integration for people with DS have become part of mainstream culture and social networks. This entailed fundamental changes in the education system as well as in popular culture with regard to DS. In particular, it was highly challenging to establish that a child with DS was above all a child, and as such, should be considered in the milieu of his/her peers. It was equally difficult to shift focus to the multitude of similarities between children with DS and other children, from the earlier, predominant focus on their differences (Zambon Hobart 1995).

The soundness of this new approach is becoming increasingly indisputable. However, data presented in this article also suggest an urgency to focus on the lack of social and health support for adults with DS.

It is possible that most people with DS in Italy would prefer to live in their original family. However, as parents age together with their DS child, they cannot be left alone to cope with the growing needs of their children. The challenge is the variability of need: individualised services and freedom of choice are necessary if home-based care is to be feasible (Brown & Brown 2009) for people with DS.

A change in public opinion and popular culture is needed to make *all* citizens visible, including those with DS. Support services have to adapt to the changing needs of people with DS. This requires that staff operators be properly trained (Jokinen & Brown 2010). Tailored support should be provided to families with DS individuals, but there is also a need for change in perceptions within communities: discrimination against people with DS was clearly evident in the data on the family quality of life survey research (Brown *et al*. 2010). In Italy, perhaps more so than in other countries, it is becoming increasingly evident that schooling and social inclusion for children alone do not guarantee a satisfactory quality of life for people with DS in adulthood. A comprehensive policy of inclusion and support should extend over the entire lifetime of people with DS.

Innovative solutions are needed for the social and medical support of people with DS with increasing age. As Anna Marchese, who founded with her husband the AIPD 35 years ago, has argued: ‘For the State and Society, our children, when they become adults, are healed: they don't need any more help’. For more than 10 years, the Fondazione Italiana Verso il Futuro has been dealing with different ‘small sheltered residential homes’ for cognitive disabilities in Rome, and diverse projects, according to the documented and individualised needs of the people with disability and their families. In order to avoid relocation at different stages of increasing need in support, programme design needs to take into account diverse needs within the same location.

Social policy more generally has to change. Piloting and testing different models of programme support in collaboration with institutions, based on data and actual needs, could provide answers to the problem of economic sustainability.
